# State of the Pupils in Injuries of the Head

**Published:** 1844-04-06

**Authors:** 


#### STATE OF THE PUPILS IN INJURIES OF THE HEAD.

Dr. Lawrie, in a paper on injuries of the head, states, that the pupils were contracted in four cases, contracted and insensible in three, dilated in two, dilated and insensible in two, sluggish in two, and in four cases one was contracted, the other dilated, and both insensible to the stimulus of light. In one case, they alternately contracted to a mere point, and dilated to a very large extent. The condition of the pupil does not uniformly correspond with the extent of the injury, nor with the situation of the extravasation; at the same time, in the majority of cases, it was dilated when the extravasation was very extensive, and almost invariably so previous to dissolution. In the cases of concussion, there was not any instance of the one pupil being dilated and the other contracted, while this symptom is noted as having occurred in one-sixth of the cases of extravasation.—*Medical Times.*

